# Walthard Cell Nests on the Serosal Surface of the Small Intestine Mimicking Metastatic Carcinoma: A Report of a First Case

**DOI:** 10.7759/cureus.112713

**Published:** 2026-07-15

**Authors:** Mariam N Albrahim, Rajan Arora, Abdulla Sultan

**Affiliations:** 1 Department of Histopathology, Sheikh Jaber Al-Ahmad Al-Sabah Hospital, Kuwait City, KWT; 2 Department of Pathology, Farwaniya Hospital, Kuwait City, KWT; 3 Department of Surgery, Farwaniya Hospital, Kuwait City, KWT

**Keywords:** metastatic mimic, peritoneal nodules, small intestine serosa, urothelial-type epithelium, walthard cell nests

## Abstract

Walthard cell nests (WCNs) are benign epithelial proliferations composed of urothelial-like transitional cells, most commonly encountered within the female reproductive tract and adjacent peritoneal surfaces. Although typically incidental findings, they may rarely occur in extragenital locations and can pose a diagnostic challenge due to their resemblance to metastatic tumors. To the best of our knowledge, following a review of the available literature, this is the first case of WCNs involving the serosal surface of the small intestine in a 58-year-old male patient with a history of urothelial carcinoma who presented with a strangulated umbilical hernia. Multiple small serosal nodules observed intraoperatively raised concern for metastatic disease. Histopathologic examination with immunohistochemistry confirmed the diagnosis of WCNs. This case expands the spectrum of reported anatomical locations of WCNs and highlights the importance of recognizing this benign entity to avoid misdiagnosis as metastatic disease.

## Introduction

Walthard cell nests (WCNs) are benign epithelial proliferations composed of transitional-type epithelium and are most commonly encountered as incidental microscopic findings. They arise predominantly within the female reproductive tract, particularly along the pelvic peritoneum, where their presence is thought to reflect mesothelial or Müllerian-derived epithelial metaplasia [1]. Although rare, WCNs have also been described in male patients, involving the epididymis and testicular parenchyma [2]. Isolated cases have been documented in unusual extragenital locations, including the vermiform appendix, mesocolon, and peritoneal diaphragm [3,4]. Despite their benign nature, WCNs may pose a diagnostic challenge because they can closely mimic epithelial neoplasms, malignant tumors, miliary tuberculosis, neuroendocrine tumors (NETs), or metastatic carcinoma depending on their location and clinical context [3]. Here, we report a case of WCNs identified on the serosal surface of the small intestine, discovered incidentally in a patient with a prior history of urothelial carcinoma, in whom the lesion initially raised concern for metastatic disease. To the best of our knowledge, this is the first reported case of WCNs occurring on the serosal surface of the small intestine, an extremely rare site not previously documented in the literature.

## Case presentation

**Figure 1:**
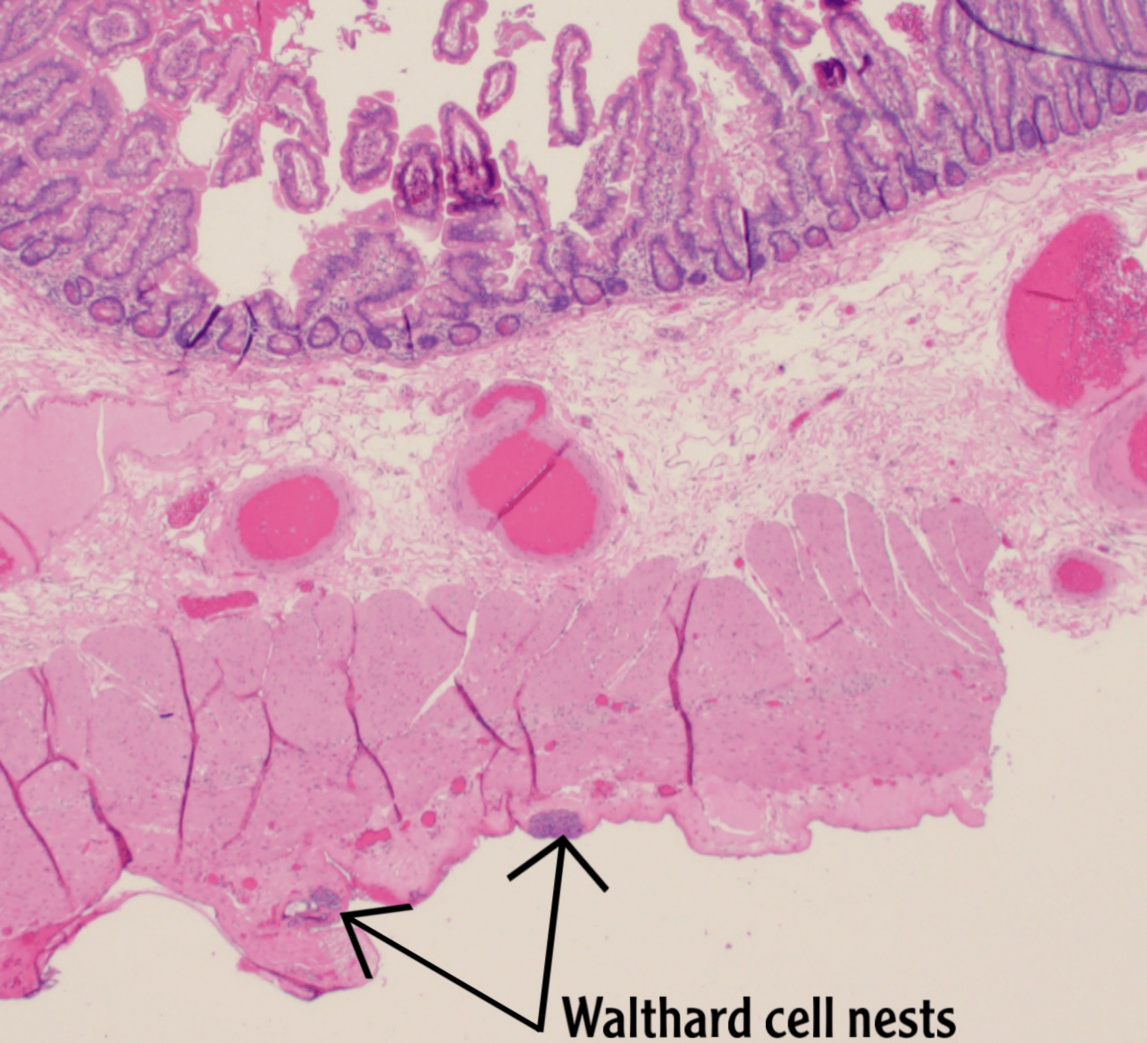
Section of the small intestine showing two nests of epithelial cells on the serosal surface (H&E, ×25) H&E: hematoxylin and eosin

**Figure 2:**
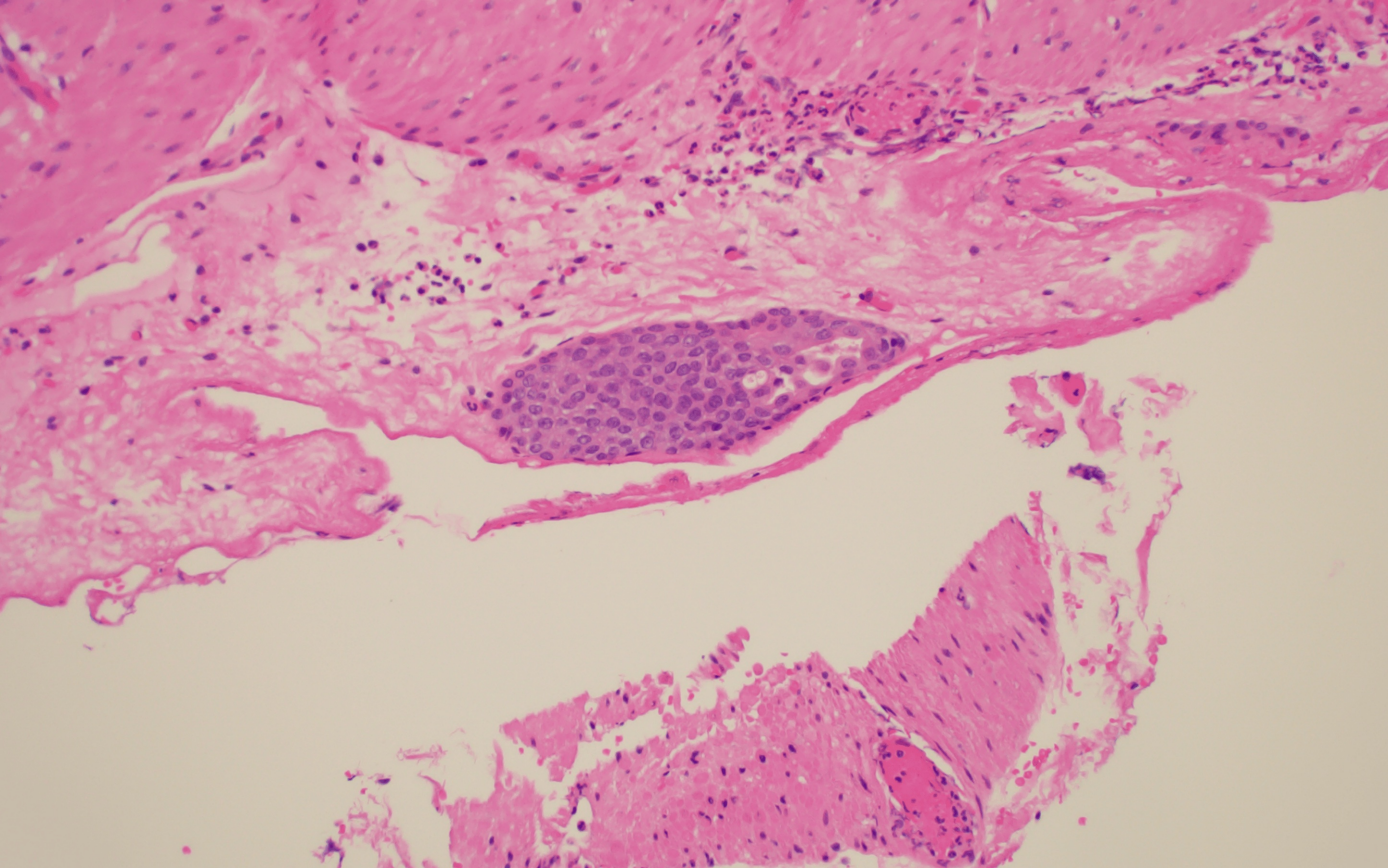
Nests of transitional-epithelial cells with no morphological atypia (H&E, ×100) H&E: hematoxylin and eosin

**Figure 3:**
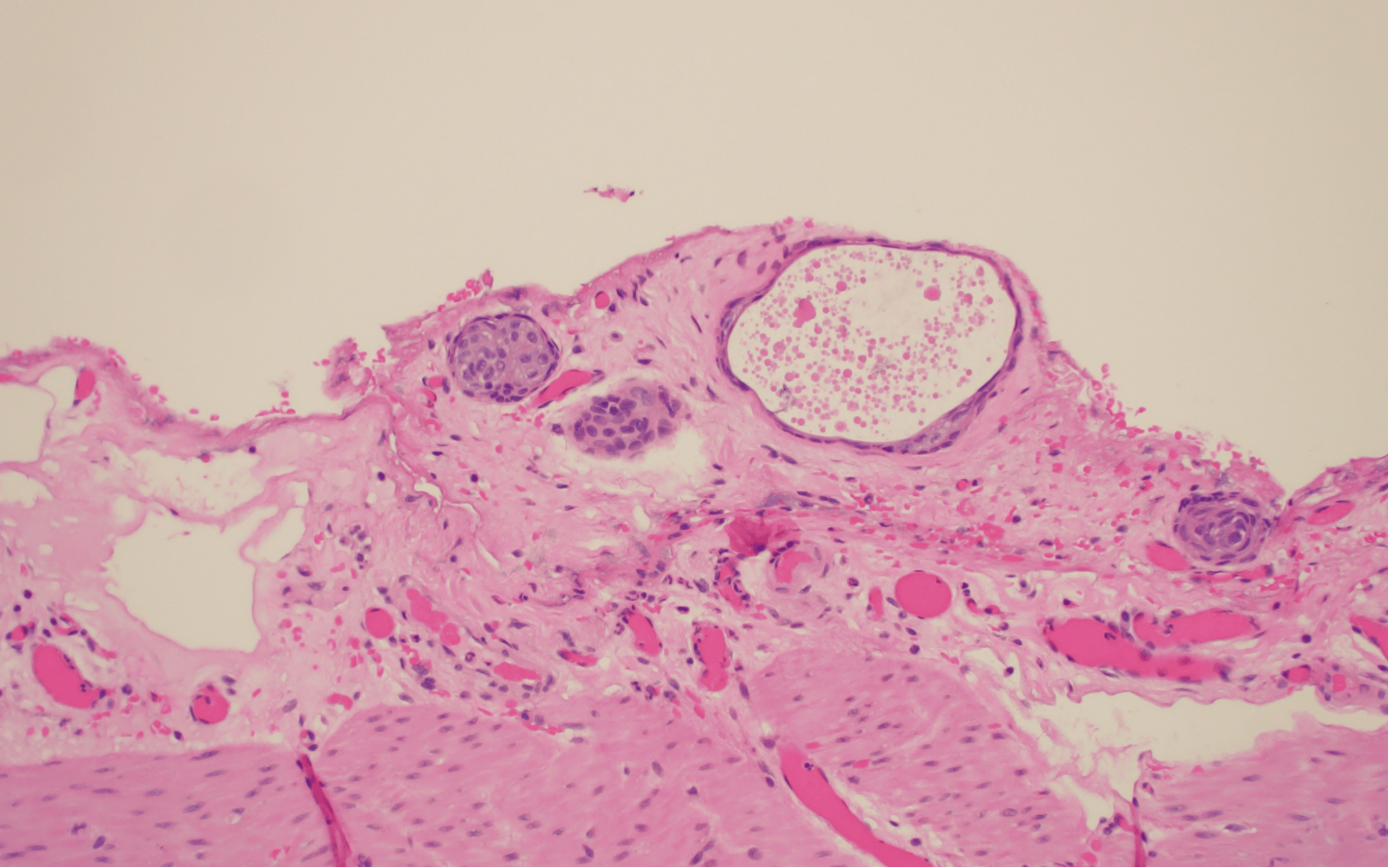
Multiple transitional-epithelial nests, some of which form cystic change (H&E, ×40) H&E: hematoxylin and eosin

A 58-year-old male patient with a prior history of urothelial carcinoma of the urinary bladder presented to the emergency department with acute, dull abdominal pain and a strangulated umbilical hernia. Abdominal ultrasound demonstrated thick-walled, dilated bowel loops consistent with an incarcerated hernia. No additional abnormalities were identified. The diagnosis and management were discussed with the patient, and emergency open surgical intervention was planned. A thorough intraoperative examination of the abdominal cavity was performed. The hernia sac was found to contain omentum and a gangrenous segment of the jejunum. Notably, multiple tiny white nodules scattered over the serosal surface of the jejunum were also observed, with a similar nodule identified on the dorsal surface of the mesentery. Segmental resection of the gangrenous jejunum and end-to-end anastomosis were performed. The postoperative course was uneventful, and the patient was discharged in stable condition. No follow-up data were available after hospital discharge, limiting the assessment of the patient's long-term outcome. Given the patient's prior history of urothelial carcinoma, these white nodules raised intraoperative concern for possible metastatic disease involving the small bowel. Upon gross pathological examination, a segment of the small intestine measuring 18 × 3.5 cm with attached mesentery was assessed. The external serosal surface appeared congested and demonstrated multiple gray-white nodules measuring 0.1-0.3 cm in diameter, while the mucosal surface was hemorrhagic. On histological examination of hematoxylin and eosin (H&E)-stained sections, mucosal necrosis with submucosal hemorrhage and edema, findings consistent with ischemic necrosis, were identified. Further evaluation of the serosal surface revealed scattered, small, solid focal cystic epithelial nests (Figures 1-3). These nests were composed of transitional-type epithelial cells with evenly spaced grooved nuclei without atypia or mitosis (Figure 2).

**Figure 4:**
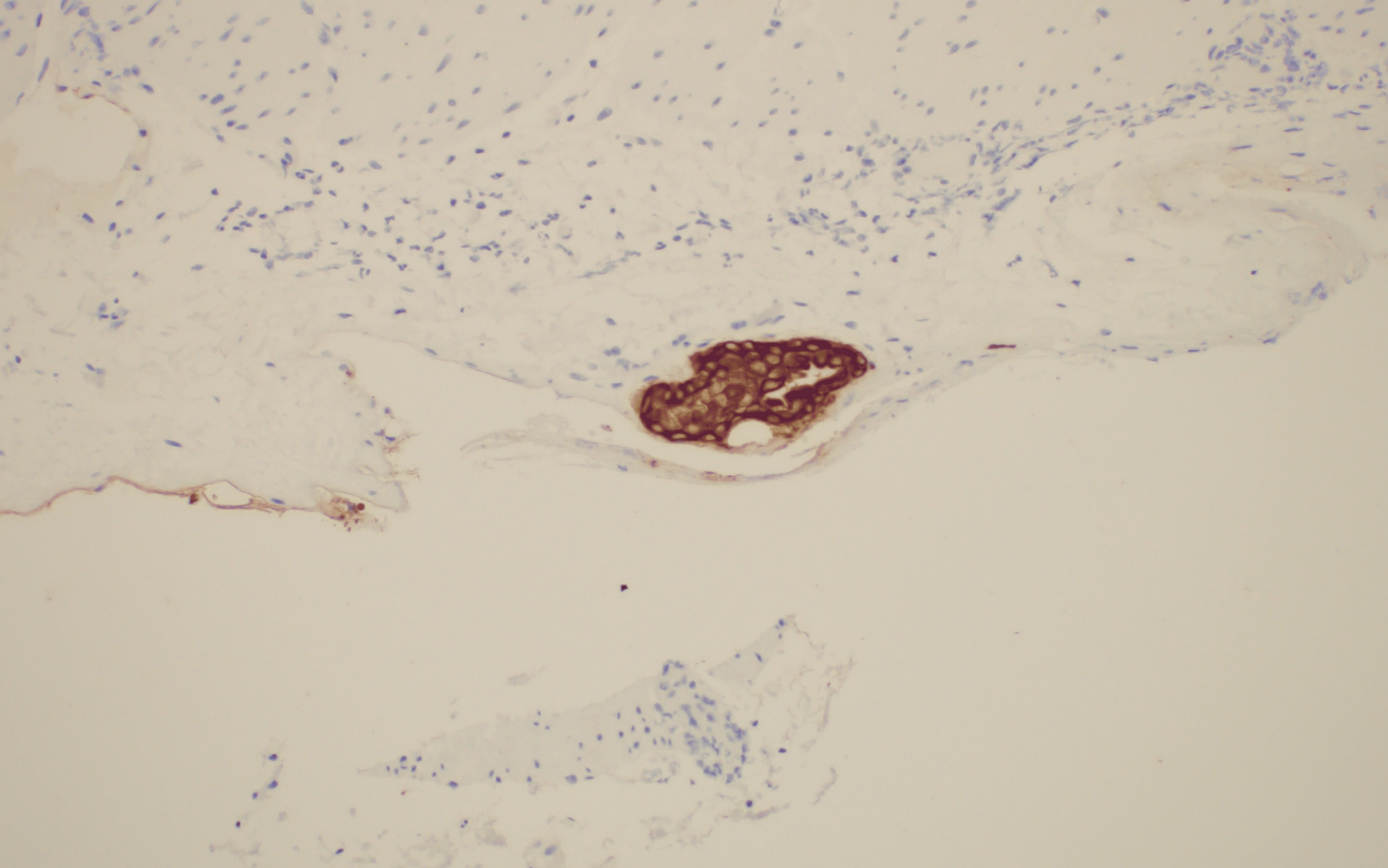
Epithelial nests expressing cytokeratin (AE1/AE3)

**Figure 5:**
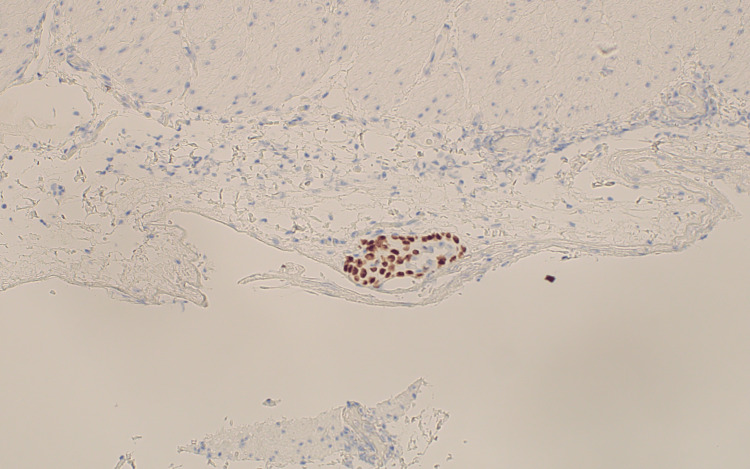
Epithelial nests expressing p63 with nuclear pattern

No associated stromal reaction or desmoplasia was identified. To further characterize these epithelial nests, immunohistochemical studies were performed. The cells showed diffuse cytoplasmic positivity for pan-cytokeratin (AE1/AE3) and nuclear positivity for p63, confirming their transitional nature, while calretinin was negative, excluding mesothelial origin (Figures 4-5).

Collectively, the clinical, gross, histological, and immunohistochemical findings were consistent with WCNs involving the serosal surface of the small intestine.

## Discussion

WCNs are uncommon benign epithelial proliferations that have been described predominantly in the female genital tract; they have rarely been reported at extragenital sites. Previously described locations include the peritoneal diaphragm, mesocolon, vermiform appendix, epididymis, tunica albuginea, testis, and spermatic cord [1-5]. Beyond its unusual anatomical location, this case has important clinical implications. In the present patient, the intraoperative finding of multiple small white nodules scattered across the serosal surface of the small intestine immediately raised suspicion for metastatic disease, particularly in light of the prior history of urothelial carcinoma. Lesions with this gross appearance may closely resemble peritoneal metastatic implants, posing a diagnostic dilemma. In addition to metastatic carcinoma, NETs represent important differential diagnoses. Both entities may demonstrate a nested growth pattern and may occur on serosal or mesenteric surfaces. However, WCNs lack the cytologic atypia and desmoplasia commonly seen in metastatic carcinoma, as well as the characteristic neuroendocrine nuclear features, including salt-and-pepper chromatin, that are hallmarks of true neuroendocrine neoplasms, allowing their distinction on H&E [5]. Miliary tuberculosis is another important differential diagnosis, as disseminated tuberculous deposits may also present as multiple small nodules. Definitive diagnosis relies on histopathologic examination rather than gross appearance alone [1]. The major differential diagnoses of WCNs are summarized in Table 1.

**Table 1:** Differential diagnoses of WCNs based on clinical, histological, and immunohistochemical features WCNs: Walthard cell nests; NET: neuroendocrine tumor; ZN: Ziehl-Neelsen; AFB: acid-fast bacilli

Feature	WCNs	Metastatic urothelial carcinoma	NET	Miliary tuberculosis	Brenner tumor
Histology	Transitional-type epithelium with bland nuclei; may show cystic change and nuclear grooves	Cytologic atypia, pleomorphism, mitoses, infiltrative growth	Uniform cells with "salt-and-pepper" chromatin	Caseating granulomas with giant cells	Nests of bland transitional-type epithelium with smooth contours embedded within a dense fibrous stroma; uniform oval nuclei with occasional nuclear grooves
Immunohistochemistry	GATA3+, pankeratin, p63	GATA3+, pankeratin, p63	Synaptophysin, chromogranin	Not applicable	GATA3+, p63
Special stain	Not applicable	Not applicable	Not applicable	ZN stain for AFB	Not applicable

Brenner tumor may be considered in the histopathologic differential diagnosis in female patients because of its shared transitional-type epithelial differentiation and nested architecture. Both lesions may demonstrate uniform nuclei, nuclear grooves, and overlapping immunophenotypic features such as aldo-keto reductase 1C3 (AKR1C3) expression [6]. However, Brenner tumors are true epithelial neoplasms characterized by a prominent fibromatous stroma and varying degrees of cytologic atypia, features that are absent in WCNs [7].

The histogenesis of WCNs remains incompletely understood. One prevailing hypothesis is that these lesions arise from the metaplastic transformation of mesothelial cells, resulting in nests of urothelial-type epithelium. This concept is supported by their frequent location along serosal and peritoneal surfaces as well as their benign, non-invasive growth pattern [1,3].

An alternative theory suggests derivation from Müllerian remnants, though this explanation is less widely supported, given the typical absence of Müllerian markers, such as paired box 8 (PAX8) and paired box 2 (PAX2), on immunohistochemical staining. The occurrence of WCNs on the serosal surface of the small intestine, as demonstrated in the present case, further substantiates mesothelial metaplasia as a potential mechanism [7].

## Conclusions

To the best of our knowledge, we report the first case of WCNs occurring on the serosal surface of the small intestine in a male patient. This case expands the spectrum of their reported anatomical distribution and highlights a potential diagnostic pitfall. Recognition of this rare presentation is important, as WCNs should be included in the differential diagnosis of multiple serosal nodules, particularly in patients with a history of malignancy, to avoid misinterpretation as metastatic disease and inappropriate tumor upstaging.
